# Mobile applications offering non-pharmacological therapies aimed at improving the quality of life of cancer patients: a scoping review

**DOI:** 10.1590/0034-7167-2024-0510

**Published:** 2025-12-08

**Authors:** Cristiano de Oliveira Ribeiro, Leonel dos Santos Silva, Lourdes Maria Rosinski Lima Gomes, Daniela Lourenço Pinto, Tereza Maria Mendes Diniz de Andrade Barroso, Luciana Puchalski Kalinke

**Affiliations:** I Universidade Federal do Paraná. Curitiba, Paraná, Brazil; II Escola Superior de Enfermagem de Coimbra. Coimbra, Portugal

**Keywords:** Mobile Applications, Complementary Therapies, Neoplasms, Quality of Life, Review., Aplicaciones Móviles, Terapias Complementarias, Neoplasias, Calidad de Vida, Revisión.

## Abstract

**Objectives:**

to map studies on mobile applications that offer non-pharmacological therapies aimed at improving the health-related quality of life of cancer patients.

**Methods:**

a scoping review was conducted based on the methodological framework of the JBI and the Preferred Reporting Items for Systematic Reviews and Meta-Analyses extension for Scoping Reviews checklist. Data search, selection, and analysis were carried out between April and May 2024 across the BVS, EMBASE, MEDLINE, PubMed, Scopus, Web of Science, and CINAHL databases.

**Results:**

seven articles published between 2018 and 2022 were included. Five studies (71.4%) were randomized clinical trials, classified as level of evidence 1B. The “Headspace” app was cited in two studies (28.6%), and mindfulness interventions were implemented in six studies (85.7%). The results indicated significant effects in reducing anxiety and depression, as well as improvements in health-related quality of life.

**Conclusions:**

the studies demonstrated that mobile applications are feasible interventions in clinical practice.

## RESUMEN

**Objetivos:**

mapear estudios sobre aplicaciones móviles que ofrecen terapias no farmacológicas dirigidas a mejorar la calidad de vida relacionada con la salud de pacientes con cáncer.

**Métodos:**

se realizó una revisión de alcance basada en el marco metodológico del JBI y en la lista de verificación *Preferred Reporting Items for Systematic Reviews and Meta-Analyses extension for Scoping Reviews*. La búsqueda, selección y análisis de los datos se llevaron a cabo entre abril y mayo de 2024 en las bases de datos BVS, EMBASE, MEDLINE, PubMed, Scopus, *Web of Science* y CINAHL.

**Resultados:**

se incluyeron siete artículos publicados entre 2018 y 2022. Cinco estudios (71,4%) fueron ensayos clínicos aleatorizados, clasificados con un nivel de evidencia 1B. La aplicación “*Headspace*” fue citada en dos estudios (28,6%) y las intervenciones de *mindfulness* se aplicaron en seis estudios (85,7%). Los resultados indicaron efectos significativos en la reducción de la ansiedad y la depresión, así como mejoras en la calidad de vida relacionada con la salud.

**Conclusiones:**

los estudios demostraron que las aplicaciones móviles son viables como intervenciones en la práctica clínica.

## INTRODUCTION

In contemporary times, the widespread adoption of technologies such as tablets and smartphones has driven the global population’s use of mobile applications, also known as apps. Conceived as sets of tools designed to perform specific tasks and functions, apps allow users to access information and knowledge without time or space constraints. In this context, this technological trend paves the way for a new form of healthcare delivery known as mHealth, in which health information is available in a timely and ubiquitous manner^(1)^.

The resources and information generated by health apps can be used to optimize outcomes and reduce health risks, as well as to understand the determinants that promote health and/or lead to illness. In this scenario, this technology emerges as a substantial alternative for effectively addressing and meeting population health needs, offering benefits such as personalization, overcoming mobility limitations, and immediate access to information, thereby enabling greater treatment engagement and contributing to the promotion of health and well-being^(2)^.

Apps, particularly those designed for cancer patients, have the potential to support healthcare professionals and patients in managing psychological distress and diagnosis, treatment planning and monitoring, information delivery and reinforcement, medication adherence, and the management of side effects^(3)^. Additionally, they enhance healthcare by serving as tools that facilitate self-care and empower patients in the self-management of clinical conditions, as they can strengthen users’ skills and confidence in managing their chronic conditions^(4)^.

The therapeutic journey of a cancer patient involves challenges encompassing physical and psychosocial aspects. From the onset of symptoms through diagnosis and treatment, cancer patients experience a complex array of emotions that affect their daily lives and perspectives on life^(5)^. In this context, the ability to self-regulate thoughts, feelings, and behaviors is often impaired, negatively impacting health-related quality of life (HRQoL)^(4)^.

The interrelation between physical and psychosocial symptoms and their impact on HRQoL highlights the importance of holistic approaches in oncology care^(4)^. This implies not only the effective clinical management of symptoms but also sensitive and comprehensive attention to the psychosocial dimensions of the patient throughout the disease trajectory^(5)^. This understanding should guide personalized, compassionate, and patient-centered care practices.

Within this context, non-pharmacological therapies (NPTs) have gained prominence as complementary alternatives for symptom self-management in cancer. According to the CEPS Platform, a major French collaborative initiative focused on the evaluation of prevention and support programs, NPTs are defined as “non-invasive and non-pharmacological interventions in human health based on science” and aim to prevent and minimize various health conditions. They involve the use of different products, methods, and techniques, based on biological mechanisms and/or psychological processes, which can significantly contribute to health and HRQoL. This French organization classifies NPTs into five categories of health interventions: physical, psychological, nutraceutical, elemental, and technological^(6)^.

The use of NPTs among cancer patients has become increasingly popular due to their broad availability and ease of practice compared to other therapies^(7)^. With the advancement of mobile health, individuals are increasingly empowered to take greater responsibility for their health and actively seek health apps and other devices to self-manage their symptoms^(8)^. Thus, NPTs integrated into mobile app technology are emerging as a promising alternative.

Although mobile apps show significant potential in symptom management and well-being promotion, evidence supporting their effectiveness remains limited. The lack of robust research in this area suggests that, to gain a comprehensive understanding of the impact of app-mediated NPTs, existing investigations must be further expanded^(9)^. In the current context, knowledge about available apps offering NPTs to cancer patients remains incipient, with a lack of data elucidating how these technologies can effectively contribute to the self-management of clinical conditions associated with cancer treatment, particularly in promoting HRQoL.

Given the above, this study is relevant as it fills a gap in the literature concerning NPT-focused apps, highlighting the need to expand the existing knowledge base by identifying the types of content and resources these tools provide.

To identify scoping reviews addressing similar objectives, a search was conducted in January 2024 in the following databases: JBI Clinical Online Network of Evidence for Care and Therapeutics (COnNECT+), Database of Abstracts of Reviews of Effects (DARE), The Cochrane Library, and the International Prospective Register of Ongoing Systematic Reviews (PROSPERO). The results indicated the absence of similar reviews on this topic.

## OBJECTIVES

To map studies on mobile applications that provide non-pharmacological therapies aimed at improving the health-related quality of life of cancer patients.

## METHODS

### Type of study

This is a scoping review registered on the Open Science Framework (https://osf.io/pcnxt/) under DOI 10.17605/OSF.IO/PCNXT. It was developed based on the methodological framework proposed by Arksey and O’Malley^(10)^, later refined by the JBI^(11)^, and structured according to the recommendations of the Preferred Reporting Items for Systematic Reviews and Meta-Analyses Extension for Scoping Reviews (PRISMA-ScR) guide^(12)^. As a scoping review, this study did not require approval from a Research Ethics Committee.

### Data source and search strategy

To formulate the research question, the “PCC” mnemonic was used: Population - Adult cancer patients; Concept - Mobile applications; Context - NPTs for HRQoL (Chart 1). Based on these definitions, the following guiding question was established: What studies on mobile applications demonstrate the use of NPTs to improve the HRQoL of adults with cancer?

**Chart 1:** Descriptors and keywords used in the search, Curitiba, Paraná, Brazil, 2024

PCC	Variables	Selected Descriptors
P *(population)*	Adult cancer patients	“*Neoplasias*” OR “*Neoplasia*” OR “*Câncer*” OR “Cancer” OR “Neoplasm” OR “Neoplasms” OR “Tumor” OR “Tumors” OR “Malignant Neoplasm” OR “Malignant Neoplasms” OR “Tumeurs” ^*^filtro: adultos
C *(concept)*	Mobile applications	“*Tecnologia Digital*” OR “Digital Technology” OR “*Aplicativos Móveis*” OR “Mobile Applications” OR “Aplicaciones Móviles” OR “Mobile Application” OR “Mobile Applications” OR “Mobile App” OR “Smartphone Apps” OR “Smartphone App”
C *(context)*	Non-pharmacological therapies for health-related quality of life	“Complementary Therapies” OR “Complementary Medicine” OR “Alternative Medicine” OR “Alternative Therapies” OR “Complementary Therapeutic Methods” OR “Alternative Medicine” OR “Complementary Therapeutic Modalities” AND “*Qualidade de Vida*” OR “*Qualidade de vida relacionada à saúde*” OR “Quality of Life” OR “Health Related Quality of Life”

*NPT - Non-Pharmacological Therapies; HRQoL - Health-Related Quality of Life.*

Data collection and selection were carried out in April 2024. Seven data sources were used: Virtual Health Library (*Biblioteca Virtual em Saúde* - BVS) Salud, EMBASE, PUBMED Central, Elsevier Scopus, Web of Science, Medical Literature Analysis and Retrieval System Online (MEDLINE), and the Cumulative Index to Nursing and Allied Health Literature (CINAHL) (accessed via the EMBASE and EBSCO platforms, respectively).

The search strategy (Chart 2) was developed in collaboration with a professional librarian. Descriptors and their alternative terms indexed in the Health Sciences Descriptors (DeCS) and Medical Subject Headings (MeSH) were used: “Digital Technology,” “Mobile Applications,” “Complementary Therapies,” “Quality of Life,” and “Neoplasms.” Additionally, the Boolean operators “AND” and “OR” were used to combine the descriptors.

**Chart 2:** Search strategy in the data sources, Curitiba, Paraná, Brazil, 2024

Data source	Search strategy
BVS	(“*Neoplasias*” OR “*Câncer*” OR “Neoplasms” OR “Tumeurs”) AND (“*Tecnologia Digital*” OR “Digital Technology”) OR (“*Aplicativos Móveis*”) OR (“Mobile Applications” OR “*Aplicaciones Móviles*”) AND (“*Qualidade de Vida*” OR “*Qualidade de vida relacionada à saúde*” OR “Quality of Life”)
EMBASE	(((‘alternative medicine’:ti,ab,kw OR ‘complementary therapeutic methods’:ti,ab,kw OR ‘alternative medicine’/exp) AND ‘neoplasm’:ti,ab,kw OR ‘neoplasm’/exp) AND ‘digital technology’:ti,ab,kw OR ‘digital technology’/exp OR ‘mobile application’:ti,ab,kw OR ‘mobile application’/exp AND ‘quality of life’:ti,ab,kw OR ‘quality of life’/exp
MEDLINE	(((‘alternative medicine’:ti,ab,kw OR ‘complementary therapeutic methods’:ti,ab,kw OR ‘alternative medicine’/exp) AND ‘neoplasm’:ti,ab,kw OR ‘neoplasm’/exp AND ‘digital technology’:ti,ab,kw OR ‘digital technology’/exp AND ‘mobile application’:ti,ab,kw OR ‘mobile application’/exp AND ‘quality of life’:ti,ab,kw OR ‘quality of life’/exp
PUBMED	(((((((((((((“complementary therapies”[MeSH Terms] OR “complementary therapies”[Title/Abstract]) OR “complementary therapeutic modalities”[Title/Abstract]) AND (“neoplasms”[MeSH Terms] OR “neoplasms”[Title/Abstract])) OR “cancer”[Title/Abstract]) AND (“digital technology”[MeSH Terms] OR “digital technology”[Title/Abstract])) OR “mobile applications”[MeSH Terms] OR “mobile applications”[Title/Abstract]) AND (“quality of life”[MeSH Terms] OR “quality of life”[Title/Abstract]))
SCOPUS	(TITLE-ABS-KEY(“Complementary Therapies” OR “Complementary Medicine” OR “Alternative Medicine” OR “Alternative Therapies”) AND TITLE-ABS-KEY(“Neoplasms” OR “Neoplasm” OR “Neoplasia” OR “Neoplasias” OR “Cancer” OR “Malignant Neoplasm”) AND TITLE-ABS-KEY(“Mobile Applications” OR “Mobile Application” OR “Mobile App” OR “Digital Technology”) AND TITLE-ABS-KEY (“Quality of Life” OR “Health Related Quality of Life”))
Web of Science	((ALL=(“Complementay Therapies” OR “Complementary Medicine” OR “Alternative Medicine” OR “Alternative Therapies”)) AND ALL=(String 3 “Neoplasms” OR “Neoplasm” OR “Neoplasia” OR “Neoplasias” OR “Cancer” OR “Malignant Neoplasm”)) AND ALL=(“Mobile Applications” OR “Mobile Application” OR “Mobile Apps” OR “Mobile App” OR “Smartphone Apps” OR “Smartphone App”) AND ALL=(“Quality of Life” OR “Health Related Quality of Life”)
CINAHL	(“*Neoplasias*” OR “*Câncer*” OR “Neoplasms” OR “Tumeurs”) AND (“*Tecnologia Digital*” OR “Digital Technology”) OR (“*Aplicativos Móveis*”) OR (“Mobile Applications” OR “*Aplicaciones Móviles*”) AND (“*Qualidade de* Vida” OR “*Qualidade de vida relacionada à saúde*” “Quality of Life”)

*BVS - Virtual Health Library (Biblioteca Virtual em Saúde).*

### Eligibility criteria

The inclusion criteria defined for this review were: studies available in full text, with no time restrictions, involving adult patients undergoing various types of cancer treatment; use of mobile applications with NPT interventions for the self-management of clinical conditions related to cancer treatment, with a focus on promoting HRQoL; randomized clinical trials or observational studies; and publications in Portuguese, Spanish, or English.

The following studies were excluded: letters to the editor, editorials, abstracts, and opinion papers; studies involving technologies intended for cancer prevention, screening, or diagnosis; those using telecommunications tools, websites, social networks, or communication platforms; studies focused on the development and validation of mobile applications; and those not centered on interventions with measurement of health-related variables.

### Study selection and data extraction

For article search and access, institutional login was used via the CAPES Journal Portal (*Periódicos da Coordenação de Aperfeiçoamento de Pessoal de Nível Superior*), accessed through the CAFe platform (*Comunidade Acadêmica Federada*), a service that simplifies access to articles through the registered university login.

During the study selection phase, a peer review process was used with the support of the web-based application Rayyan QCRI^®^ for screening and managing systematic reviews. Initially, one researcher was responsible for exporting the search result files from each database into the tool, followed by identification and removal of duplicate studies.

Screening of the articles was conducted independently by two researchers, based on the reading of titles and abstracts. In cases of discrepancies or doubts about an article’s relevance to the study scope, a third researcher was consulted to resolve the disagreements.

Both researchers proceeded with the independent selection of eligible studies, resulting in few disagreements. To resolve divergences, the researchers engaged in a discussion of the studies and reached a consensus based on the study’s objectives and predefined inclusion criteria. This process led to the definition of the final sample of selected studies.

**Figure 1:**
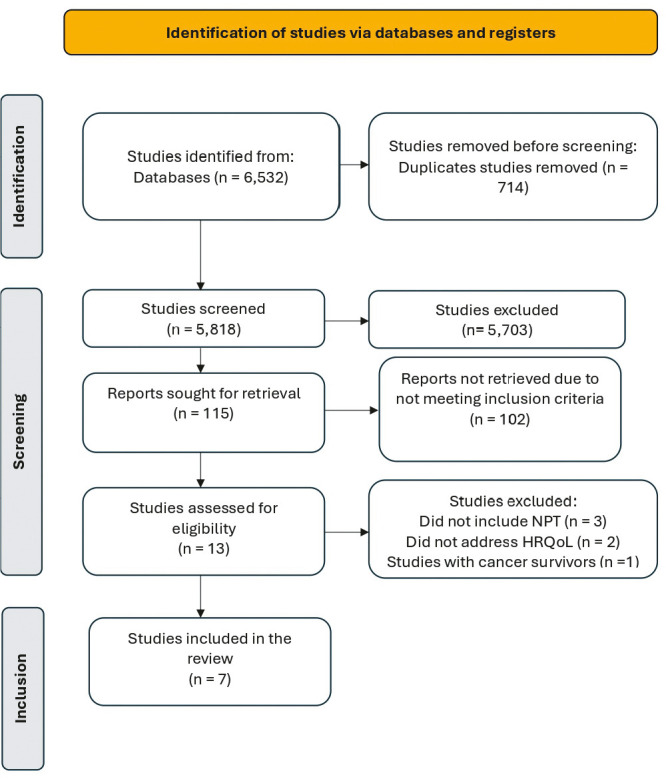
Study identification and inclusion process - Preferred Reporting Items for Systematic Reviews and Meta-Analyses (PRISMA) flow diagram, Curitiba, Paraná, Brazil, 2024

Data extraction was performed without disagreement between reviewers. It was not necessary to involve the third researcher or to contact the primary authors regarding the data. Figure 1 outlines the results of each stage of the review process, in accordance with the PRISMA Flow Diagram model^(13)^.

### Data analysis

The analysis of the studies was conducted in May 2024. Data were extracted, organized, and synthesized into tables using Microsoft Excel®, following a structured format that included the following categories: authors and year of publication, country of publication, study objective, and study type. Each study was critically evaluated for its level of evidence (LoE) and grade of recommendation, in accordance with the standards established by the Oxford Centre for Evidence-Based Medicine^(14)^. The data were then organized and synthesized in the following order: application, intervention, population and sample, and outcomes.

## RESULTS

### Study selection

The initial search identified a total of 6,532 published studies. After duplicate removal, 5,818 studies were selected for preliminary screening, which involved identifying the term “socioemotional well-being” in the title, abstract, and keywords. Of these, 5,703 studies did not meet this selection criterion and were therefore excluded.

Following the initial screening of titles, abstracts, and keywords, 115 studies were pre-selected for more detailed evaluation. Of these, 102 were excluded for not meeting the established inclusion criteria. The remaining 13 studies were considered eligible and underwent full-text review. Of these, six were excluded-three for not including NPTs, two for not addressing HRQoL, and one for involving cancer survivors. Thus, seven studies were deemed relevant and constituted the final sample.

The seven studies included in the final sample were published between 2018 and 2022: three (42.8%) in 2018^(15-17)^, one (14.3%) in 2020^(18)^, and three (42.8%) in 2022^(19-21)^. Among the selected studies, four (57.1%)^(15,17-19)^ were conducted in the United States of America (USA), followed by one study each in Spain (14.3%)^(21)^, Norway (14.3%)^(20)^, and Switzerland (14.3%)^(16)^.

In terms of methodology, five studies (71.4%) were randomized clinical trials^(17-21)^, one (14.3%) was a cohort study^(15)^, and one (14.3%) was a prospective observational study^(16)^. Regarding the level and quality of evidence, five (71.4%) were classified as 1B with a grade A recommendation^(17-21)^.

As for the types of NPTs and the objectives of the selected studies: four studies (57.1%) evaluated the effectiveness of apps featuring mindfulness training programs^(15,17,18,21)^; one (14.3%) analyzed the effectiveness of a mindfulness program combined with guided relaxation; one (14.3%) assessed the efficacy of cognitive-behavioral exercises including mindfulness and guided imagery^(20)^; and one (14.3%) evaluated the effectiveness of a Tai Chi mobile application^(19)^ (Chart 3).

**Chart 3:** Characterization of the studies included in the scoping review, Curitiba, Paraná, Brazil, 2024

ID	Reference	Country	Objective	Level of Evidence / Grade of Recommendation	Study Type
E1^(20)^	BØRØSUND et al., 2022	Norway	To evaluate the effectiveness of StressProffen in cancer patients.	1B/A	RCT
E2^(19)^	GAO, RYU e CHEN et al., 2022	USA	To investigate the effects of a Tai Chi mobile app and a Facebook program on stress and HRQoL in breast cancer patients.	1B/A	RCT
E3^(21)^	GONZÁLEZ et al., 2022	Spain	To compare the effectiveness of a mobile app-based mindfulness training with usual treatment.	1B/A	RCT
E4^(15)^	KUBO et al., 2018	USA	To evaluate the feasibility and acceptability of a mobile app-based mindfulness intervention for cancer patients undergoing chemotherapy and their primary caregivers.	2B/B	Pilot
E5^(18)^	PUZIA et al., 2020	USA	To conduct an exploratory secondary analysis of the effects of a smartphone app on depression, anxiety, sleep disorders, and HRQoL in patients with myeloproliferative neoplasms.	1B/A	RCT
E6^(17)^	ROSEN et al., 2018	USA	To evaluate the effectiveness of mindfulness training delivered via a mobile app on the HRQoL of women with breast cancer.	1B/A	RCT
E7^(16)^	MIKOLASEK, WITT e BARTH, 2018	Switzerland	To evaluate the feasibility of a mindfulness and relaxation app for cancer patients.	2B/B	Observational

*ID - identification; USA - United States of America; RCT - Randomized Controlled Trial.*

Six studies (85.7%)^(15,16,18-21)^ examined the effects of the interventions on anxiety, depression, and HRQoL. Of these, two (28.6%)^(19,20)^ included an analysis of stress, and one (14.3%)^(21)^ addressed satisfaction and pain. Additionally, one study (14.3%)^(17)^ explored mindfulness, pain, and HRQoL.

Among the analyzed applications, *Headspace* was the most frequently used, being mentioned in two studies (28.6%)^(15,17)^. Mindfulness-based interventions were employed in six studies (85.7%), encompassing various types and stages of cancer^(15-18,20,21)^. The results demonstrated significant effects in reducing anxiety and depression, along with improvements in HRQoL.

All studies in the sample used quantitative questionnaires to measure patient-reported outcomes, employing validated instruments specifically designed to assess the target outcomes, with a primary focus on HRQoL.

Chart 4 presents synthesized information from the studies, including the name of the application, description of the intervention, study population and sample, analyzed variables, instruments used, and the main outcomes reported.

**Chart 4:** Summary of apps, interventions, and main results evidenced in the studies included in the scoping review, Curitiba, Paraná, Brazil, 2024

ID	App	Description	Intervention and Application	Population and Sample	Outcomes or Advanced Variables	Results
E1^(20)^	StressProffen	App with ten modules containing explanatory materials and educational exercises for cognitive-behavioral stress management.	Guidance and management of stress and anger, self-care, meditation, mindfulness, guided imagery, assertiveness, communication, and goal setting. App used until completion of the ten modules, with follow-up phone calls.	Multiple cancers and stages (n=172) under any type of treatment.	Stress (PSS), anxiety and depression (HADS), fatigue (SRF-18), and HRQoL (HRQOL/RAND 36).	Reduction in stress (p < 0.001), depression (p = 0.003), fatigue (p = 0.002), and improvement in HRQoL (six of eight domains, p ≤ 0.015).
E2^(19)^	7 Minute Chi	App for Tai Chi beginners. Teaches correct breathing and posture for each movement.	Tai Chi. App used three times a day for at least five days a week.	Advanced breast cancer (n=35) in curative treatment.	Stress (PSS), anxiety, depression, sleep disturbance, and HRQoL (PROMIS).	Improved HRQoL in the mental health domain (p = 0.05).
E3^(21)^	En Calma e Quirófano	App for mindfulness exercises with two training programs: short and long.	Mindfulness. Daily app use until completion of the training program.	Colorectal cancer (n=102) in surgical treatment.	Anxiety and depression (HADS), HRQoL (WHOQOL-BREF), pain (VAS), and satisfaction (CSQ-8).	Reduced anxiety and depression at two points (B = -0.2; 95% CI between 8.8 and 9.2); no effect on HRQoL.
E4^(15)^	Headspace	App with instructions for meditation and mindfulness practice.	Mindfulness. App used for eight weeks with basic and specific modules.	Cancer patients in chemotherapy (n=28) and caregivers (n=14).	Distress (TD), fatigue (BFI), anxiety and depression (HADS), sleep disturbances (PSQI), and HRQoL (PROMIS).	Reduction in distress levels (p < 0.01), anxiety (p < 0.05); improved mental health (p < 0.01) and sleep quality (p < 0.05).
E5^(18)^	Calm	Meditation, sleep improvement, and guided relaxation app.	Mindfulness. Daily use for ten minutes over four weeks.	Myeloproliferative neoplasm (n=80) with no treatment specified.	Anxiety, depression, sleep disturbances, and HRQoL (PROMIS).	Reduced anxiety (p < 0.001) and depression (p < 0.001).
E6^(17)^	Headspace	App with instructions for meditation and mindfulness practice.	Mindfulness. Used for eight weeks with modules released weekly.	Breast cancer (n=102) under various treatment types.	HRQoL (FACT-B), mindfulness (MASS), and pain (BPI).	Improved HRQoL (p < 0.01).
E7^(16)^	CanRelax	App with three exercises: mindfulness, guided imagery, and progressive muscle relaxation guided by audio.	Mindfulness and guided relaxation. Daily app use.	Multiple cancers and stages (n=100), treatment not specified.	Distress (TD), HRQoL (FACT-G), anxiety and depression (HADS).	Feasible intervention, with acceptable adherence and positive feedback.

*Notes: ID: identification; App: application; HRQoL: health-related quality of life; STAI: State-Trait Anxiety Inventory; RSES: Rosenberg Self-Esteem Scale; HADS: Hospital Anxiety and Depression Scale; WHOQOL-BREF: World Health Organization Quality of Life - Brief Version; VAS: Visual Analog Scale (for pain); CSQ-8: Client Satisfaction Questionnaire; PSQI: Pittsburgh Sleep Quality Index; PROMIS: Patient-Reported Outcomes Measurement Information System; FACT-B: Functional Assessment of Cancer Therapy - Breast Version 4; MASS: Mindful Attention Awareness Scale; BPI: Brief Pain Inventory - Short Form; DASS: Depression, Anxiety and Stress Scale; FACT-G: Functional Assessment of Cancer Therapy - General; PSS: Perceived Stress Scale; SRF-18: Self-Regulatory Fatigue; DT: Distress Thermometer; BFI: Brief Fatigue Inventory; HRQOL: Health-Related Quality of Life; VAS: Visual Analog Scale.*

## DISCUSSION

This review aimed to map, in the literature, studies involving mobile applications that provide NPTs aimed at improving the HRQoL of cancer patients. Accordingly, the included studies seek to expand the understanding of available resources to support patients during treatment while highlighting the importance of technology use in the oncology context. Understanding the effectiveness and scope of these interventions can provide crucial information for healthcare teams and patients, contributing to improved well-being and HRQoL.

Regarding the level of evidence of the studies included in this review, the predominance of robust methodologies may be justified by the complexity of the topic under investigation. In this sense, producing knowledge using rigorous methods in the field of oncology becomes essential. This approach aims to provide reliable information and applicable science for clinical care, supporting patient-centered decisions aligned with best practices in an effective and safe manner^(22)^.

All studies employed quantitative questionnaires to collect patient self-reported data, using instruments specifically designed to measure subjective health outcomes, particularly HRQoL. Although health assessment typically relies on objective metrics, the subjective perceptions provided by individuals through these instruments play a crucial role in enabling a deeper understanding of their health condition. This holistic approach, combined with clinical outcome assessments, should be valued in clinical practice and contributes to a comprehensive and accurate understanding of the patient experience^(23)^.

Adopting a holistic perspective enables a broad and integrative understanding of the illness process as a whole, rather than viewing it solely as a system of organs. By considering the physical, mental, and spiritual aspects of health, this approach promotes not only physical healing but also overall well-being and balance. This integrated view, when combined with conventional treatment and the use of NPTs, can lead to significant improvements in patient survival and quality of life^(24)^.

In this review, the main NPTs provided by mobile applications were mindfulness^(6,13,14,16,18,19)^, guided imagery relaxation^(6)^, and Tai Chi^(17)^. These interventions were offered either independently or in combination with cognitive-behavioral exercises or relaxation techniques, thereby expanding the therapeutic options available to patients.

Mindfulness, one of the highlighted NPTs, is a mind-body approach defined as moment-to-moment awareness with a nonjudgmental attitude^(15,21)^. Gradually integrated into cancer treatment, it has emerged as a promising strategy for symptom management^(25,26)^ and for coping with health-related stress, with the goal of minimizing psychological harm and promoting physical and emotional well-being. By enabling patients to use this intervention, the aim is to encourage them to adopt a proactive stance toward their symptoms and promote effective self-management of both psychological and physical conditions^(25)^.

In recent years, there have been increasing efforts to investigate the beneficial effects of mindfulness-based interventions^(26)^. In this context, systematic reviews with meta-analyses^(25,27)^ have been conducted to evaluate the effects of these interventions among cancer patients. The results indicate significant improvements in anxiety, depression, and fatigue levels, with positive implications for HRQoL.

Moreover, the benefits of mindfulness are evident in the mapped studies^(15-18,20,21)^ included in this review, which highlight the positive effects of app-guided interventions for cancer patients. The main reported benefits include reduced symptoms of anxiety and depression^(15,20,21)^, fatigue and stress^(20)^, improved mental health^(15,19)^ and sleep quality^(15)^, all of which positively impact HRQoL^(17,20,21)^. These findings demonstrate the intervention’s viability, acceptable adherence, and favorable patient feedback^(16)^.

Regarding the use of online mindfulness, a systematic review of randomized clinical trials (n = 10), conducted with cancer patients (n = 962), demonstrated the feasibility, acceptability, and efficacy of this intervention. The results showed significant reductions in distress (p = 0.008), depression (p = 0.03), stress (p = 0.05), sleep disturbances (p = 0.04), and improvements in HRQoL (p = 0.03). However, no significant effects were found for anxiety, mindfulness, rumination, fear of cancer recurrence, fatigue, or post-traumatic stress^(28)^.

Special attention is commonly given to the HRQoL of cancer patients, as the therapeutic process often negatively impacts physical, social, emotional, and functional well-being. The aggressive nature of the disease and its associated treatments can result in cognitive impairment, depression, and physical limitations, which manifest as psychological distress. These adversities, combined with misconceptions about the disease, can hinder treatment adherence and effectiveness^(23)^.

In addition to the positive outcomes in HRQoL associated with the use of NPTs, the studies included in this review indicate a significant link between HRQoL and factors such as psychological stress, depression, or anxiety, and show that these variables may negatively affect both the quality of life and mortality of cancer patients. This was demonstrated in a rapid review of systematic reviews (n = 12), which suggested an association between chronic stress, depression, anxiety, or stressful life events and both the incidence of and mortality from cancer^(29)^. This underscores the importance of further research-including studies employing technology and NPTs-aimed at addressing and mitigating this issue.

Breast cancer emerged as the most frequently studied neoplasm among the included studies^(17,19)^. One study^(30)^ suggests that women diagnosed with breast cancer face challenges in effectively managing symptoms such as pain, depression, and fatigue at home. The adoption of healthy lifestyle habits and regular physical activity can reduce risk and improve breast cancer prognosis, which in turn positively influences HRQoL.

Tai Chi stands out as a beneficial exercise for promoting health and well-being among women with breast cancer^(19)^. Originating in China, Tai Chi is a mind-body NPT recognized as a form of moving meditation. It is based on breathing and gentle body movements, promoting a meditative mental state that can significantly contribute to overall well-being^(31)^.

A review study^(31)^, which included 210 systematic reviews of randomized controlled trials (with a median of 750 participants per review), concluded that Tai Chi offers multidimensional benefits across a wide range of conditions and morbidities, including physical, psychological, and HRQoL-related aspects. According to the authors, in the context of women with breast cancer, the effects of Tai Chi were found to be balanced or equivalent to those of the control group. However, in some studies, methodological limitations prevented definitive conclusions. Nonetheless, clinically significant improvements were observed in fatigue and pain.

While clinical evidence is essential for evaluating any health-supporting tool or intervention, it is equally important to consider time and human behavior when assessing health applications. Given that these apps are used continuously and independently by patients, it remains uncertain how much time is required for them to produce tangible effects and foster effective changes in symptom self-management^(8)^.

In the context of multifactorial issues-especially following the COVID-19 pandemic-thousands of people are experiencing stress and anxiety. Many mobile applications can offer self-guided techniques for managing distress in adults, facilitated through smartphones. Among them is *Headspace*, one of the most widely used health apps across different platforms, which has surpassed 70 million downloads in over 190 countries^(32)^.

In this review, based on the results of the included studies, it was observed that app-guided NPTs yielded positive outcomes in HRQoL and well-being among the studied population^(15,17-20)^. A systematic review^(4)^ of randomized clinical trials (12 studies, 2,627 participants) analyzed the effects of digital health interventions on self-management skills in cancer patients. Eight of the 12 studies revealed statistically significant improvements in variables such as enhanced self-efficacy, knowledge, and proactivity regarding health care. Additionally, significant improvements were observed in HRQoL, as well as in emotional and social functioning.

A key consideration lies in the substantial impact of cancer on patients’ mental well-being-a phenomenon that encompasses multiple emotional and psychological dimensions which, if not properly managed, may compromise HRQoL. The illness experience evokes a complex range of conflicting emotions, shaped by apprehension over disease progression and hope for recovery. This whirlwind of individual experiences triggers not only psychological and emotional instability but also broader implications for mental well-being, with significant fluctuations in patients’ outlook on life and overall HRQoL^(5)^.

In some studies, emotional and psychological well-being emerged as a relevant domain in the assessment of HRQoL, with additional analyses examining the impact of interventions on stress, anxiety, and depression^(15,16,18-21)^. Approximately 30% of cancer patients experience symptoms of anxiety or depression, which negatively affect both health status and HRQoL^(21)^. Consideration of these aspects highlights the broad and multifaceted nature of the quality-of-life concept and contributes to a more comprehensive and holistic understanding of patient care^(5)^.

The inclusion of additional articles in the discussion, beyond those identified in the final sample of this scoping review, is justified by the need to adequately contextualize the study’s findings within a broader scientific framework. By incorporating new studies, it was possible to enrich the interpretation of the results and reflect the complexity of the research field.

### Study limitations

Given the high number of studies not retrieved, it is important to acknowledge this limitation when discussing the results. The exclusion of certain studies may be attributed to restrictions in the consulted databases, the absence of specific descriptors, the inclusion criteria adopted, or potential indexing issues. The exclusion of grey literature represents another limitation, as the authors chose to focus exclusively on studies published in scientific journals, which ensures greater validity and credibility of the presented data.

### Contributions to the field of Nursing

This review contributes to the health field-particularly nursing-by presenting information on various NPTs mediated by technology. These therapies have proven to be effective and safe and can be adopted by healthcare teams as a complement to conventional treatment. Their goal is to promote the self-management of clinical conditions related to cancer treatment and to support the HRQoL of oncology patients.

## CONCLUSIONS

This scoping review made it possible to map studies on the use of mobile applications offering NPTs for cancer patients aimed at improving HRQoL. These findings may assist in understanding the relevance of such technologies in the self-management of clinical conditions related to cancer treatment, with a focus on promoting HRQoL in both scientific and professional contexts.

This topic has generated growing interest among both the general population and healthcare professionals. However, there is still a limited number of studies evaluating the effectiveness of app-mediated NPTs, highlighting the need for continued research and development in this field.

The findings revealed that NPTs delivered via smartphone applications have the potential to improve the HRQoL of cancer patients and influence variables such as stress, anxiety, and depression. This suggests that such interventions may contribute positively as a complement to conventional cancer treatment, promoting the well-being of this population.

## Data Availability

The research data are available only upon request.
