# Supplementary material for: Mobile applications offering non-pharmacological therapies aimed at improving the quality of life of cancer patients: a scoping review
Source: Rev Bras Enferm. 2025 Dec 8;78(Suppl 4):e20240510. doi: 10.1590/0034-7167-2024-0510 (PMC12700572; doi:10.1590/0034-7167-2024-0510)
Supplement: Translation

# Translation: Aplicativos com terapias não farmacológicas direcionados à qualidade de vida de pacientes com câncer: *scoping review*

**DOI:** 10.1590/0034-7167-2024-0510pt

**Authors:** Cristiano de Oliveira Ribeiro, Leonel dos Santos Silva, Lourdes Maria Rosinski Lima Gomes, Daniela Lourenço Pinto, Tereza Maria Mendes Diniz de Andrade Barroso, Luciana Puchalski Kalinke

**Affiliations:** I Universidade Federal do Paraná. Curitiba, Paraná, Brasil; II Escola Superior de Enfermagem de Coimbra. Coimbra, Portugal

**Keywords:** Aplicativos Móveis, Terapias Complementares, Neoplasias, Qualidade de Vida, Revisão.

## Abstract

**Objetivos:**

mapear estudos sobre aplicativos móveis que oferecem terapias não farmacológicas direcionadas à qualidade de vida relacionada à saúde de pacientes com câncer.

**Métodos:**

revisão de escopo com base no referencial metodológico do JBI e no *checklist Preferred Reporting Items for Systematic Reviews and Meta-Analyses extension for Scoping Reviews*. As buscas, seleção e análise dos dados ocorreram entre abril e maio de 2024, nas bases BVS, EMBASE, MEDLINE, PubMed, Scopus, *Web of Science* e CINAHL.

**Resultados:**

foram incluídos sete artigos, publicados entre 2018 e 2022. Cinco (71,4%) eram ensaios clínicos randomizados, com nível de evidência 1B. O aplicativo “*Headspace*” foi mencionado em dois estudos (28,6%) e a intervenção de *mindfulness* foi aplicada em seis estudos (85,7%). Os resultados mostraram efeitos significativos na redução da ansiedade, da depressão e melhorias na qualidade de vida relacionada à saúde.

**Conclusões:**

os estudos demonstraram que os aplicativos são factíveis como intervenção na prática clínica.

## INTRODUÇÃO

Na contemporaneidade, é possível constatar a disseminação de tecnologias, como *tablets* e *smartphones*, que impulsionaram, entre a população mundial, o uso de aplicativos móveis, também conhecidos como *apps*. Concebidos como conjuntos de ferramentas projetadas para executar tarefas e funções específicas, os *apps* possibilitam o acesso do usuário à informação e ao conhecimento, sem restrição de tempo e espaço. Nessa conjuntura, essa tendência tecnológica pavimenta o caminho para uma nova forma de assistência conhecida como *mHealth*, na qual as informações de saúde estão disponíveis de forma oportuna e onipresente^(1)^.

Os recursos e informações gerados por *apps* de saúde podem ser utilizados para otimização dos resultados e redução dos riscos em saúde, bem como para compreensão dos fatores determinantes que promovem a saúde e/ou que levam à doença. Neste cenário, esta tecnologia emerge como uma alternativa substancial para abordar de maneira eficaz e atender às demandas de saúde da população, ao oferecer benefícios como pessoalidade, quebra de limitação de mobilidade e acesso imediato a informações, fato que possibilita o engajamento no tratamento e auxilia na promoção da saúde e do bem-estar^(2)^.

Os *apps*, em especial os destinados aos pacientes com câncer, têm o potencial de auxiliar profissionais de saúde e pacientes no diagnóstico e sofrimento psicológico associado, planejamento e acompanhamento do tratamento, fornecimento e fortalecimento de informações, apoio à adesão medicamentosa e no controle dos efeitos colaterais^(3)^. Além disso, aprimoram os cuidados de saúde ao representar um dos meios para facilitar o autocuidado e capacitar a autogestão de condições clínicas entre os pacientes, uma vez que podem fortalecer a habilidade e a confiança do usuário no gerenciamento de sua condição crônica^(4)^.

O percurso terapêutico do paciente com câncer envolve desafios que abrangem aspectos físicos e psicossociais. Desde o início das manifestações no organismo, diagnóstico e tratamento, pacientes com câncer vivenciam uma complexidade de sentimentos que refletem no cotidiano e em suas perspectivas de vida^(5)^. Nessa diretiva, a capacidade de autorregular pensamentos, sentimentos e comportamentos é frequentemente prejudicada e reflete negativamente na qualidade de vida relacionada à saúde (QVRS)^(4)^.

A inter-relação entre os sintomas físicos e psicossociais e seu impacto na QVRS ressalta a importância de abordagens holísticas no cuidado oncológico^(4)^. Isso implica não apenas na gestão clínica eficaz dos sintomas, mas também na atenção sensível e abrangente às dimensões psicossociais do paciente durante o processo de enfrentamento da doença^(5)^. Essa compreensão deve guiar práticas de cuidado personalizadas, compassivas e centradas no paciente.

Nesse contexto, as terapias não farmacológicas (TNF) ganham destaque como alternativa complementar para autogestão dos sintomas do câncer. De acordo com a Plataforma CEPS, uma importante iniciativa colaborativa francesa focada na avaliação de programas de prevenção e suporte, a TNF “é uma intervenção não invasiva e não farmacológica na saúde humana baseada na ciência” e tem como objetivo prevenir e minimizar diferentes agravos. Consiste no uso de diferentes produtos, métodos e técnicas, com mecanismos de ação biológicos e/ou processos psicológicos, que podem contribuir significativamente na saúde e QVRS. Esta organização francesa classifica a TNF em cinco categorias de intervenções em saúde: físicas, psicológicas, nutracêuticas, elementares e tecnológicas^(6)^.

O emprego de TNF em pacientes com câncer tem se tornado popular, incitado por sua ampla disponibilidade e pela facilidade de prática em comparação com outras terapias^(7)^. Com o avanço da saúde móvel, os indivíduos estão cada vez mais capacitados a assumir maior responsabilidade pela sua saúde e buscam com entusiasmo *apps* de saúde e outros dispositivos para autogestão dos seus sintomas^(8)^. Assim, as TNF integradas à tecnologia dos *apps* móveis emergem como uma alternativa próspera.

Apesar de os *apps* móveis demonstrarem potencial significativo na gestão de sintomas e na promoção do bem-estar, as evidências que sustentam sua eficácia permanecem limitadas. A carência de pesquisas robustas nesse domínio sugere que, para se alcançar uma compreensão abrangente do impacto das TNF mediadas por *apps*, é imperativo aprofundar as investigações já existentes^(9)^. No contexto atual, o conhecimento acerca dos *apps* disponíveis com TNF oferecidas a pacientes com câncer ainda é incipiente, caracterizando-se pela insuficiência de dados que elucidem como essas tecnologias podem efetivamente contribuir para a autogestão de condições clínicas associadas ao tratamento do câncer, com particular ênfase na promoção da QVRS.

Diante do exposto, este estudo é relevante por preencher uma lacuna literária acerca de *apps* sobre TNF, destacando-se a necessidade de ampliar o conhecimento existente, de forma a conhecer conteúdos e recursos apresentados.

Para identificar revisões de escopo que abordassem objetivos semelhantes, foi realizada uma busca em janeiro de 2024 nas seguintes bases de dados: *JBI Clinical Online Network of Evidence for Care and Therapeutics* (*COnNECT+*), *Database of Abstracts of Reviews of Effects* (*DARE*), *The Cochrane Library* e *International Prospective Register of Ongoing Systematic Reviews* (*PROSPERO*). Os resultados indicaram a ausência de revisões semelhantes sobre essa temática.

## OBJETIVOS

Mapear estudos sobre aplicativos móveis que disponibilizam terapias não farmacológicas direcionadas a qualidade de vida relacionada à saúde de pacientes com câncer.

## MÉTODOS

### Tipo de estudo

Trata-se de uma revisão de escopo registrada no *Open Science Framework* (https://osf.io/pcnxt/) sob identificação DOI 10.17605/OSF.IO/PCNXT, desenvolvida com base no referencial metodológico proposto por Arksey e O’Malley^(10)^, posteriormente aprimorado pelo JBI^(11)^ e estruturada com base nas recomendações do guia *Preferred Reporting Items for Systematic Reviews and Meta-Analyses Extension for Scoping Reviews* (PRISMA-ScR)^(12)^. Por ser um estudo do tipo revisão de escopo, não foi necessária a apreciação pelo Comitê de Ética em Pesquisa.

### Fonte de dados e estratégia de busca

Para a formulação da questão de pesquisa, utilizou-se o mnemônico “PCC”: População - Pacientes adultos com câncer; Conceito - Aplicativos móveis; Contexto - TNF para a QVRS (Quadro 1). Com base nessas definições, foi estabelecida a seguinte questão norteadora: *Quais estudos sobre aplicativos móveis demonstram o uso de TNF para QVRS de adultos com câncer?*

**Quadro 1:** Descritores e palavras-chave utilizadas na busca, Curitiba, Paraná, Brasil, 2024

PCC	Variáveis	Descritores selecionados
P *(population)*	Pacientes adultos com câncer	*“*Neoplasias*” OR “*Neoplasia*” OR “*Câncer*” OR “Cancer” OR “Neoplasm” OR “Neoplasms” OR “*Tumor*” OR “Tumors” OR “Malignant Neoplasm” OR “Malignant Neoplasms” OR “Tumeurs”* ^*^filtro: adultos
C *(concept)*	Aplicativos móveis	*“*Tecnologia Digital*” OR “Digital Technology” OR “*Aplicativos Móveis*” OR “Mobile Applications” OR “Aplicaciones Móviles” OR “Mobile Application” OR “Mobile Applications” OR “Mobile App” OR “Smartphone Apps” OR “Smartphone App”*
C *(context)*	Terapias não farmacológicas para qualidade de vida relacionada à saúde	*“Complementary Therapies” OR “Complementary Medicine” OR “Alternative Medicine” OR “Alternative Therapies” OR “Complementary Therapeutic Methods” OR “Alternative Medicine” OR “Complementary Therapeutic Modalities” AND “*Qualidade de Vida*” OR “*Qualidade de vida relacionada à saúde*” OR “Quality of Life” OR “Health Related Quality of Life”*

*TNF - Terapias não farmacológicas; QVRS - Qualidade de vida relacionada à saúde.*

O levantamento e seleção dos dados ocorreram em abril de 2024. Foram utilizadas sete fontes de dados: Biblioteca Virtual em Saúde (BVS), EMBASE, PUBMED Central, *Elsevier Scopus*, Web Of Science e *Medical Literature Analysis and Retrieval System Online* (MEDLINE) e *Cumulative Index to Nursing and Allied Health Literature* (CINAHL) (via plataforma EMBASE e EBSCO, respectivamente).

A estratégia para a busca (Quadro 2) foi desenvolvida em conjunto com um profissional bibliotecário. Utilizou-se os descritores e seus termos alternativos indexados ao Descritores em Ciências da Saúde (DeCS) e ao *Medical Subject Headings* (MESH): *“Digital Technology”, “Mobile Applications”, “Complementary Therapies”, “Quality of Life” e “Neoplasms”*. Adicionalmente, empregaram-se os operadores booleanos “AND” e “OR” para o cruzamento dos descritores.

**Quadro 2:** Estratégia de busca nas fontes de dados, Curitiba, Paraná, Brasil, 2024

Fonte de dados	Estratégia de busca
BVS	(“Neoplasias” *OR* “Câncer” *OR* “*Neoplasms*” *OR* “*Tumeurs*”) *AND* (“Tecnologia Digital” *OR* “*Digital Technology*”) *OR* (“Aplicativos Móveis”) *OR (“Mobile Applications” OR “Aplicaciones Móviles”) AND (“*Qualidade de Vida*” OR “*Qualidade de vida relacionada à saúde*” OR “Quality of Life”)*
EMBASE	*(((‘alternative medicine’:ti,ab,kw OR ‘complementary therapeutic methods’:ti,ab,kw OR ‘alternative medicine’/exp) AND ‘neoplasm’:ti,ab,kw OR ‘neoplasm’/exp) AND ‘digital technology’:ti,ab,kw OR ‘digital technology’/exp OR ‘mobile application’:ti,ab,kw OR ‘mobile application’/exp AND ‘quality of life’:ti,ab,kw OR ‘quality of life’/exp*
MEDLINE	*(((‘alternative medicine’:ti,ab,kw OR ‘complementary therapeutic methods’:ti,ab,kw OR ‘alternative medicine’/exp) AND ‘neoplasm’:ti,ab,kw OR ‘neoplasm’/exp AND ‘digital technology’:ti,ab,kw OR ‘digital technology’/exp AND ‘mobile application’:ti,ab,kw OR ‘mobile application’/exp AND ‘quality of life’:ti,ab,kw OR ‘quality of life’/exp*
PUBMED	*(((((((((((((“complementary therapies”[MeSH Terms] OR “complementary therapies”[Title/Abstract]) OR “complementary therapeutic modalities”[Title/Abstract]) AND (“neoplasms”[MeSH Terms] OR “neoplasms”[Title/Abstract])) OR “cancer”[Title/Abstract]) AND (“digital technology”[MeSH Terms] OR “digital technology”[Title/Abstract])) OR “mobile applications”[MeSH Terms] OR “mobile applications”[Title/Abstract]) AND (“quality of life”[MeSH Terms] OR “quality of life”[Title/Abstract]))*
SCOPUS	*(TITLE-ABS-KEY(“Complementary Therapies” OR “Complementary Medicine” OR “Alternative Medicine” OR “Alternative Therapies”) AND TITLE-ABS-KEY(“Neoplasms” OR “Neoplasm” OR “Neoplasia” OR “Neoplasias” OR “Cancer” OR “Malignant Neoplasm”) AND TITLE-ABS-KEY(“Mobile Applications” OR “Mobile Application” OR “Mobile App” OR “Digital Technology”) AND TITLE-ABS-KEY (“Quality of Life” OR “Health Related Quality of Life”))*
Web of Science	*((ALL=(“Complementay Therapies” OR “Complementary Medicine” OR “Alternative Medicine” OR “Alternative Therapies”)) AND ALL=(String 3 “Neoplasms” OR “Neoplasm” OR “Neoplasia” OR “Neoplasias” OR “Cancer” OR “Malignant Neoplasm”)) AND ALL=(“Mobile Applications” OR “Mobile Application” OR “Mobile Apps” OR “Mobile App” OR “Smartphone Apps” OR “Smartphone App”) AND ALL=(“Quality of Life” OR “Health Related Quality of Life”)*
CINAHL	*(“*Neoplasia*s” OR “*Câncer*” OR “Neoplasms” OR “Tumeurs”) AND (“*Tecnologia Digital*” OR “Digital Technology”) OR (“Aplicativos Móveis”) OR (“Mobile Applications” OR “Aplicaciones Móviles”) AND (“*Qualidade de Vida*” OR “*Qualidade de vida relacionada à saúde*” “Quality of Life”)*

*BVS - Biblioteca virtual em saúde.*

### Critérios de elegibilidade

Os critérios de inclusão delimitados para esta revisão foram: estudos acessíveis na íntegra, sem restrição temporal, com pacientes adultos em diferentes modalidades de tratamento do câncer; uso de aplicativos móveis com intervenção de TNF para a autogestão de condições clínicas relacionadas ao tratamento do câncer, com foco na promoção da QVRS; ensaios clínicos ou estudos observacionais; idiomas português, espanhol e inglês.

Foram excluídos os seguintes estudos: cartas ao editor, editoriais, resumos e estudos de opinião; os que utilizaram tecnologias destinadas à prevenção, rastreamento e diagnóstico do câncer; com ferramentas de telecomunicações, sites, redes sociais, plataformas de comunicação; os destinados ao desenvolvimento e validação de aplicativos móveis; e os que não se concentraram na intervenção com mensuração de variáveis de saúde.

### Seleção de estudos e extração de dados

Para a busca e leitura dos artigos, utilizou-se o acesso institucional na plataforma de Periódicos da Coordenação de Aperfeiçoamento de Pessoal de Nível Superior (CAPES), via acesso à plataforma CAFe (Comunidade Acadêmica Federada), serviço que simplifica a disponibilização e o acesso a artigos por meio do login utilizado da respectiva universidade cadastrada.

Na fase de seleção de estudos, por meio de revisão por pares, empregou-se o aplicativo *web* para seleção e apoio à construção de revisões *Rayyan QCRI^®^*. Inicialmente, um dos pesquisadores encarregou-se da exportação dos arquivos com os resultados da busca em cada base de dados para a ferramenta, conduzindo posteriormente à identificação e exclusão dos estudos duplicados.

A triagem dos artigos foi conduzida por dois pesquisadores de maneira independente, com base na leitura dos títulos e resumos. Em caso de discrepâncias na seleção ou dúvidas sobre a relevância dos artigos para o escopo do estudo, um terceiro pesquisador seria consultado para resolver as divergências.

Ambos os pesquisadores procederam com a seleção individual dos estudos elegíveis, resultando em poucas discordâncias na escolha. Para dirimir divergências, os pesquisadores engajaram-se em um diálogo sobre os estudos e solucionaram as discrepâncias com base nos objetivos do estudo e nos critérios de inclusão pré-estabelecidos. Esse processo conduziu à definição da amostra final de estudos selecionados.

Os dados foram obtidos sem discordância entre os revisores, que não consideraram a inclusão do terceiro pesquisador ou a necessidade de contatar os autores primários sobre os dados. A Figura 1 explicita os resultados de cada etapa da análise, de acordo com o modelo PRISMA *Flow Diagram* ^(13)^.

**Figura 1:** Processo de identificação e inclusão dos estudos - *Preferred Reporting Items for Systematic Reviews and Meta-Analyses* (PRISMA) *diagram flow*, Curitiba, Paraná, Brasil, 2024

### Análise dos dados

A análise dos estudos ocorreu em maio de 2024. Os dados foram extraídos, organizados e sintetizados em tabelas no *Microsoft Excel* ^®^, seguindo uma disposição estruturada, que incluiu as seguintes categorias: autores e ano de publicação, local de publicação, objetivo do estudo, tipo de estudo. Cada estudo foi submetido a uma avaliação criteriosa em relação ao nível de evidência (NE) e grau de recomendação, de acordo com os padrões estabelecidos pelo *Oxford Centre Evidence-Based Medicine* ^(14)^. Em seguida, foram organizados e sintetizados na seguinte ordem: aplicativo, intervenção, população e amostra, e desfecho.

## RESULTADOS

### Seleção dos estudos

A busca inicial de dados identificou um total de 6.532 estudos publicados. Após a remoção de duplicatas, 5.818 estudos foram selecionados para uma triagem preliminar com leitura de títulos e resumos. Desse total, 5.703 estudos não atenderam a esse critério de seleção e, portanto, foram descartados.

A partir da análise dos títulos, resumos, e descritores inicial 115 estudos foram pré-selecionados para uma avaliação mais detalhada. Desses, 102 foram excluídos por não cumprirem os critérios de inclusão estabelecidos. Os 13 estudos restantes foram considerados elegíveis e passaram por uma avaliação completa, por meio da leitura integral do texto. Desses, seis foram excluídos (três por não incluírem TNF, dois por não abordarem a QVRS e um por envolver sobreviventes de câncer). Assim, sete estudos foram considerados relevantes e constituíram a amostra final.

Os sete estudos que compuseram a amostra foram publicados entre 2018 e 2022: três (42,8%) em 2018^(15-17)^, um (14,3%) em 2020^(18)^ e três (42,8%) em 2022^(19-21)^. Dos sete selecionados, quatro (57,1%)^(15,17-19)^ foram realizados nos Estados Unidos da América (EUA), seguidos por Espanha (14,3%)^(21)^, Noruega (14,3%)^(20)^ e Suíça (14,3%)^(16)^ com um estudo.

Em relação a metodologia empregada nos estudos, cinco (71,4%) foram ensaios clínicos randomizados^(17-21)^, um (14,3%) estudo de coorte^(15)^ e um (14,3%) estudo observacional prospectivo^(16)^. Quanto ao nível e a qualidade de evidência dos artigos, cinco (71,4%) foram 1B com grau de recomendação A^(17-21)^.

Quanto ao tipo de TNF e os objetivos dos estudos selecionados: quatro (57,1%) avaliaram a eficácia de aplicativos com programa de treinamento *mindfulness* ^(15,17,18,21)^, um (14,3%) analisou a eficácia de programa de treinamento *mindfulness* associado relaxamento guiado, um (14,3%) averiguou a eficácia de exercícios cognitivos comportamentais como *mindfulness* e imagens guiadas^(20)^, e um (14,3%) avaliou a eficácia de um aplicativo móvel de *Tai Chi* ^(19)^ (Quadro 3).

**Quadro 3:** Caracterização dos estudos incluídos na revisão de escopo, Curitiba, Paraná, Brasil, 2024

ID	Referência	País	Objetivo	Nível de Evidência/ Grau de Recomendação	Tipo de Estudo
E1^(20)^	BØRØSUND et al., 2022	Noruega	Avaliar a eficácia do StressProffen em pacientes com câncer.	1B/A	ECR
E2^(19)^	GAO, RYU e CHEN et al., 2022	EUA	Investigar os efeitos de um app móvel de *Tai Chi* e de um programa do Facebook sobre o estresse e a QVRS em pacientes com câncer de mama.	1B/A	ECR
E3^(21)^	GONZÁLEZ et al., 2022	Espanha	Comparar a eficácia de um treinamento de *mindfulness* por meio de um app móvel e o tratamento usual.	1B/A	ECR
E4^(15)^	KUBO et al., 2018	EUA	Avaliar a viabilidade e aceitabilidade de intervenção *mindfulness* por meio de um app móvel para pacientes com câncer em tratamento quimioterápico e seus cuidados principais.	2B/B	Piloto
E5^(18)^	PUZIA et al., 2020	EUA	Realizar uma análise exploratória e secundária dos efeitos de um app para *smartphones* na depressão, ansiedade, distúrbios do sono e QVRS em pacientes com neoplasia mieloproliferativa.	1B/A	ECR
E6^(17)^	ROSEN et al., 2018	EUA	Avaliar a eficácia do treinamento de *mindfulness* fornecido por app móvel na QVRS de mulheres com câncer de mama.	1B/A	ECR
E7^(16)^	MIKOLASEK, WITT e BARTH, 2018	Suiça	Avaliar a viabilidade de um app de *mindfulness* e relaxamento para pacientes com câncer.	2B/B	Observacional

*ID - identificação; EUA - Estados Unidos da América; ECR - Ensaio Clínico Randomizado.*

Seis estudos (85,7%)^(15,16,18-21)^, investigaram os impactos da intervenção na ansiedade, na depressão e na QVRS. Dentro desse conjunto, dois (28,6%)^(19,20)^ incluíram a análise do estresse e um (14,3%)^(21)^ da satisfação e dor. Ademais, um (14,3%)^(17)^ explorou a atenção plena, dor e a QVRS.

Entre os aplicativos analisados, o Headspace foi o mais utilizado, sendo mencionado em dois estudos (28,6%)^(15,17)^. Já as intervenções baseadas em *mindfulness* foram aplicadas em seis estudos (85,7%), abrangendo diferentes tipos e estágios de câncer^(15-18,20,21)^. Os resultados demonstraram efeitos significativos na redução da ansiedade e da depressão, além de melhorias na QVRS.

Todos os estudos da amostra empregaram questionários quantitativos para medida de resultados relatados pelo paciente, com instrumentos validados e específicos para mensuração dos desfechos, com enfoque principal na QVRS.

O Quadro 4 apresenta as informações sintetizadas dos estudos, como: nome do aplicativo, descrição da intervenção, população estudada e amostra, variáveis analisadas, instrumentos utilizados e os principais desfechos evidenciados nos estudos.

**Quadro 4:** Síntese dos aplicativos, intervenções e os principais resultados evidenciados nos estudos incluídos na revisão de escopo, Curitiba, Paraná, Brasil, 2024

ID	App	Descrição	Intervenção e aplicação	População e amostra	Desfechos ou variáveis avançadas	Resultado
E1^(20)^	StressProffen	App com dez módulos com materiais explicativos e exercícios educacionais de gerenciamento cognitivo-comportamental e estresse.	Orientação e gestão do estresse e raiva, autocuidado, meditação, *mindfulness*, imagens guiadas, assertividade, comunicação e estabelecimento de metas. Uso do app até conclusão dos dez módulos com ligações telefônicas para acompanhamento.	Múltiplos cânceres e estágios (n=172) em quaisquer tipos de tratamento.	Estresse (PSS), ansiedade e depressão (HADS), fadiga (SRF-18) e QVRS (HRQOL/RAND 36).	Redução do estresse (p < 0,001), depressão (p = 0,003), fadiga (p = 0,002), e maior QVRS (seis dos oito domínios, p ≤ 0,015).
E2^(19)^	7 Minute Chi	App para iniciantes em *Tai Chi*. Ensina respiração e postura corretas para cada movimento.	*Tai Chi*. Uso do app três vezes ao dia por no mínimo cinco dias da semana.	Mama avançado (n=35) em tratamento curativo.	Estresse (PSS), ansiedade, depressão, distúrbio do sono e QVRS (PROMIS).	Maior QVRS no domínio da saúde mental (p = 0,05).
E3^(21)^	En Calma e Quirófano	App para exercícios de atenção plena com dois programas de treinamento: curto e longo.	*Mindfulness*. Uso do app diário até conclusão do programa de treinamento.	Colorretal (n=102) em tratamento cirúrgico.	Ansiedade e depressão (HADS), QVRS (WHOQOL-BREF), dor (VAS) e satisfação (CSQ-8).	Redução da ansiedade e depressão em dois momentos (B=-0,2; IC95% entre 8,8 e 9,2); sem efeito na QVRS.
E4^(15)^	Headspace	App com instruções para prática de meditação e atenção plena.	*Mindfulness*. Uso do aplicativo durante oito semanas com módulo básico e específicos.	Câncer em tratamento quimioterápico (n=28) e cuidadores (n=14).	Sofrimento (TD), fadiga (BFI), ansiedade e depressão (HADS), distúrbios do sono (PSQI) e QVRS (PROMIS).	Redução dos níveis de sofrimento (p <0,01), ansiedade (p <0,05); maior saúde mental (p <0,01) e qualidade de sono (p <0,05).
E5^(18)^	Calm	App de meditação, melhora do sono e relaxamento guiado.	*Mindfulness*. Uso do app diário por dez min. por quatro semanas.	Neoplasia mieloproliferativa (n=80) sem especificação de tratamento.	Ansiedade, depressão, distúrbios do sono e QVRS (PROMIS).	Redução da ansiedade (p <0,001) e depressão (p <0,001).
E6^(17)^	Headspace	App com instruções para prática de meditação e atenção plena.	*Mindfulness*. Uso do app por oito semanas com liberação de módulos a cada semana.	Mama (n=102) em quaisquer tipos de tratamento.	QVRS (FACT-B), atenção plena (MASS) e dor (BPI).	Maior QVRS (p <0,01).
E7^(16)^	CanRelax	App com três exercícios: *mindfulness*, imagens guiadas e relaxamento muscular progressivo guiado por áudio.	*Mindfulness* e relaxamento guiado. Uso do app diário.	Múltiplos cânceres e estágios (n=100) sem especificação de tratamento.	Sofrimento (TD), QVRS (FACT-G), ansiedade e depressão (HADS).	Intervenção viável, com adesão aceitável e *feedback* positivo.

*ID: identificação; App: aplicativo; QVRS: qualidade de vida relacionada à saúde; IDATE: Inventário de Ansiedade Traço-Estado; RSES: Escala de Autoestima Rosenberg; HADS: Questionário de Ansiedade e depressão; WHOQOL- BREF: Qualidade de vida; EVA: Escala de dor; CSQ-8: questionário de satisfação; PSQI: Índice de Qualidade do Sono de Pittsburg; PROMIS: Sistema de Medição de Resultados Relatados por Pacientes: FACT-B: Functional Assesment of Cancer Therapy - Breast Version 4; MASS: Mindful Attention Awareness Scale; BPI: Inventário Breve de Dor - Formulário Curto; DASS: Questionário de auto-relato de depressão, ansiedade e estresse; FACT-G: Functional Assessment of Cancer Therapy-General; PSS: Perceived Stress Scale; SRF-18: Self-Regulatory Fatigue; TD: Termômetro de Distress; BFI: Brief. Fatigue Inventory; HRQOL: Health Related Quality of Life; VAS: Visual Analog Scale.*

## DISCUSSÃO

Esta revisão teve como objetivo mapear, na literatura, estudos com aplicativos móveis que disponibilizam TNF, direcionadas à QVRS, utilizados por pacientes com câncer. Desse modo, os estudos que a integram visam a ampliar a compreensão sobre os recursos disponíveis para o suporte aos pacientes no tratamento, destacando, ao mesmo tempo, a importância do uso de tecnologias no contexto da oncologia. Ao compreender a eficácia e a abrangência dessas intervenções, é possível fornecer informações cruciais para equipes de saúde e pacientes, os quais podem auxiliar na melhoria do bem-estar e da QVRS.

Em relação ao nível de evidência dos estudos incluídos nesta revisão, a predominância de metodologias robustas pode ser justificada pela complexidade do tema pesquisado. Nesse sentido, torna-se crucial a produção de conhecimento com métodos rigorosos na área da oncologia. Essa abordagem visa disponibilizar informações confiáveis e ciência aplicável ao cuidado clínico, subsidiando decisões centradas no paciente e alinhadas às melhores práticas, de forma eficaz e segura^(22)^.

Todos os estudos empregaram questionários quantitativos para coletar informações autorrelatadas pelos pacientes, com instrumentos específicos para mensuração de desfechos subjetivos referentes à saúde, com foco na QVRS. Embora a avaliação em saúde exija métricas objetivas, as percepções subjetivas fornecidas pelo indivíduo por meio desses instrumentos desempenham um papel crucial ao permitir uma compreensão mais profunda da sua condição de saúde. Essa abordagem holística, aliada à avaliação dos resultados clínicos, deve ser valorizada na prática clínica e contribui para uma compreensão abrangente e precisa da experiência do paciente^(23)^.

Adotar uma perspectiva holística possibilita uma compreensão ampla e integral do processo de adoecimento como um todo e não apenas como um sistema de órgãos. Ao considerar os aspectos físicos, mentais e espirituais da saúde, promove-se não apenas a cura física, mas também o bem-estar geral e o equilíbrio da saúde. Essa visão integrada, aliada ao tratamento convencional e ao uso de TNF, pode resultar em melhorias significativas na sobrevida e na qualidade de vida dos pacientes^(24)^.

Nesta revisão, destacam-se como principais TNF disponibilizadas por aplicativos o *mindfulness* ^(6,13,14,16,18,19)^, o relaxamento por imagem guiada^(6)^ e o *Tai Chi* ^(17)^. Essas intervenções foram ofertadas de maneira independente ou ainda combinadas com exercícios cognitivo-comportamentais ou técnicas de relaxamento, ampliando as opções terapêuticas disponíveis aos pacientes.

O *mindfulness*, uma das TNF destacadas, é uma abordagem mente-corpo definida como consciência momento a momento com uma atitude de não julgamento^(15,21)^. Gradualmente integrado ao tratamento do câncer, emerge como uma abordagem promissora para a gestão dos sintomas^(25,26)^ e do estresse relacionado à saúde, com o objetivo de minimizar os danos psicológicos e promover o bem-estar físico e emocional. Ao capacitar o paciente a utilizar essa intervenção, objetiva-se encorajá-lo a adotar uma postura proativa em relação aos sintomas e promover a autogestão eficaz dos sintomas psicológicos e físicos^(25)^.

Nos últimos anos, houve crescentes esforços para investigar os efeitos benéficos de intervenções que incorporam o *mindfulness* ^(26)^. Nesse cenário, revisões sistemáticas com meta-análise^(25,27)^ foram conduzidas para analisar os efeitos da intervenção entre pacientes com câncer. Os resultados indicam melhorias significativas nos níveis de ansiedade, depressão e fadiga, com implicações positivas na QVRS.

Outrossim, os benefícios do *mindfulness* são evidentes no mapeamento dos estudos^(15-18,20,21)^ desta revisão, que destacam os efeitos positivos da intervenção guiada por aplicativos para pacientes com câncer. Entre os principais efeitos benéficos estão a redução de sintomas de ansiedade e depressão^(15,20,21)^, fadiga e estresse^(20)^, melhoria na saúde mental^(15,19)^ e no sono^(15)^, com reflexo positivo na QVRS^(17,20,21)^, mostrando-se como uma intervenção viável, com adesão aceitável e retorno positivo dos pacientes^(16)^.

Em relação ao uso de *mindfulness* na modalidade *on-line*, uma revisão sistemática de ensaios clínicos randomizados (n = 10), conduzidos em pacientes com câncer (n = 962), destaca a viabilidade, aceitabilidade e eficácia dessa intervenção. Os resultados mostraram redução significativa no sofrimento (p = 0,008), depressão (p = 0,03), estresse (p = 0,05), distúrbios do sono (p = 0,04) e melhoria da QVRS (p = 0,03). Embora não tenha sido evidenciada significância em relação à ansiedade, atenção plena, ruminação, medo da recorrência do câncer, fadiga e estresse pós-traumático^(28)^.

Comumente, uma atenção especial é dada à QVRS de pacientes com câncer, uma vez que o transcurso terapêutico impacta negativamente o bem-estar físico, social, emocional e funcional. A natureza agressiva da enfermidade e suas terapias associadas podem resultar em comprometimento cognitivo, depressão e limitações físicas, os quais se refletem em angústia psicológica. Tais adversidades, somadas a percepções equivocadas sobre a doença, podem prejudicar a adesão ao tratamento e sua eficácia^(23)^.

Além dos resultados positivos na QVRS associados ao uso de TNF, os estudos incluídos nesta revisão apontam um vínculo significativo entre a QVRS e fatores como estresse psicológico, depressão ou ansiedade, e mostram que essas variáveis podem interferir negativamente na vida e mortalidade de pacientes com câncer. Tal fato foi evidenciado por uma revisão rápida de revisões sistemáticas (n = 12), que sugere uma associação entre estresse crônico, depressão, ansiedade ou eventos estressantes da vida, tanto em termos de incidência quanto de mortalidade por câncer^(29)^. Destaca-se a importância de investigações, inclusive com o uso de tecnologias e de TNF, com vistas a abordar e minimizar essa problemática.

O câncer de mama emergiu como a neoplasia mais frequente entre os estudos analisados^(17,19)^. O estudo^(30)^ sugere que pacientes diagnosticadas com câncer de mama enfrentam desafios na gestão eficaz de sintomas como dor, depressão e fadiga em seus lares. A adoção de hábitos de vida saudáveis e a prática de atividade física podem reduzir o risco e melhorar o prognóstico do câncer de mama, o que se reflete positivamente na QVRS.

O *Tai Chi* se destaca como um exercício benéfico para promover a saúde e o bem-estar em mulheres com câncer de mama^(19)^. Originário da China, o *Tai Chi* é uma TNF mente-corpo, reconhecida como uma forma de meditação em movimento. Baseia-se na respiração e em movimentos suaves do corpo, proporcionando um estado mental meditativo que pode contribuir significativamente para o bem-estar geral^(31)^.

Estudo de revisão^(31)^, que abrangeu 210 revisões sistemáticas de ensaios clínicos controlados (com uma mediana de 750 participantes por revisão), concluiu que o *Tai Chi* oferece benefícios multidimensionais para uma ampla variedade de condições e morbidades, incluindo aspectos físicos, psicológicos e de QVRS. De acordo com os autores, no contexto de mulheres com câncer de mama, os efeitos do *Tai Chi* foram considerados equilibrados ou equivalentes aos do grupo controle. No entanto, em alguns estudos, as fragilidades metodológicas impediram conclusões precisas. Ainda assim, melhorias clinicamente significativas foram observadas para fadiga e dor.

Embora a evidência clínica seja essencial para a avaliação de qualquer instrumento ou intervenção de auxílio à saúde, é igualmente importante considerar o tempo e o comportamento humano na avaliação de aplicativos de saúde. Dado que esses aplicativos são utilizados contínua e exclusivamente pelos pacientes, permanece incerto quanto tempo é necessário para que produzam efeitos tangíveis e promovam mudanças efetivas na autogestão dos sintomas^(8)^.

Associadas às questões multifatoriais, especialmente após a pandemia de COVID-19, milhares de pessoas enfrentam estresse e ansiedade. Muitos aplicativos móveis podem oferecer técnicas autodirigidas e de gestão do sofrimento para indivíduos adultos, as quais são possibilitadas pelos *smartphones*, dentre eles o *Headspace*, um dos aplicativos de saúde mais difundidos nas diferentes plataformas. Ele ultrapassou os 70 milhões de downloads em mais de 190 países^(32)^.

Nesta revisão, com base nos resultados dos estudos, foi possível observar que as TNF guiadas via aplicativo obtiveram resultados positivos na QVRS e no bem-estar da população estudada^(15,17-20)^. Uma revisão sistemática^(4)^, com ensaios clínicos randomizados (12 estudos, 2.627 participantes), analisou o efeito de intervenções digitais em saúde nas habilidades de autogestão em pacientes com câncer. Oito dos 12 estudos revelaram melhoria estatisticamente significativa nas variáveis analisadas, como aprimoramento da autoeficácia, conhecimento e proatividade sobre os cuidados com a saúde. Adicionalmente, observaram-se melhorias significativas na QVRS, função emocional e social.

Uma consideração essencial recai sobre os impactos substanciais do câncer no bem-estar mental dos pacientes, um fenômeno que abarca diversas dimensões emocionais e psicológicas, as quais, se não forem adequadamente gerenciadas, podem comprometer a QVRS. A experiência da enfermidade evoca uma complexidade de sentimentos opostos no indivíduo, delineados pela apreensão diante da possível progressão da doença e pela esperança de alcançar a cura. Esse turbilhão de acontecimentos singulares desencadeia não apenas instabilidades psicológicas e emocionais, mas também implicações no bem-estar mental, com oscilações significativas na perspectiva de vida do paciente e em sua QVRS^(5)^.

Foi observado, em alguns estudos, que o bem-estar emocional e psicológico emergiu na avaliação da QVRS, sendo analisado, adicionalmente, o impacto da intervenção no estresse, na ansiedade e na depressão^(15,16,18-21)^. Trinta por cento dos pacientes com câncer enfrentam sintomas de ansiedade ou depressão, os quais exercem impacto negativo na saúde e na QVRS^(21)^. A consideração desses aspectos enfatiza a natureza ampla e multifacetada do conceito de qualidade de vida e contribui para uma compreensão mais abrangente e holística do cuidado^(5)^.

Justifica-se a inclusão de novos artigos na discussão, além daqueles identificados na amostra final desta revisão de escopo, pela necessidade de contextualizar adequadamente os achados do estudo em um cenário mais amplo de produção científica. Ao incorporar novos estudos, foi possível contextualizar os achados e refletir a complexidade do campo de estudo.

### Limitações do estudo

Considerando o elevado número de estudos não recuperados, é relevante reconhecer essa limitação na discussão dos resultados. A exclusão de certos estudos pode ser atribuída a restrições nas bases de dados consultadas, à ausência de descritores específicos, aos critérios de inclusão adotados ou a possíveis falhas na indexação dos estudos. A não inclusão da literatura cinzenta representa outra limitação, uma vez que os autores optaram por focar exclusivamente em estudos publicados em revistas científicas, o que permite maior validade e credibilidade dos dados apresentados.

### Contribuições para a área da Enfermagem

Esta revisão contribui para a área da saúde, sobretudo para a enfermagem, ao apresentar informações e diferentes TNF mediadas por tecnologias. Essas terapias mostram-se eficazes e seguras, podendo ser adotadas pela equipe de saúde como complemento ao tratamento tradicional, com o objetivo de promover a autogestão de condições clínicas relacionadas ao tratamento do câncer e contribuir para a QVRS dos pacientes com câncer.

## CONCLUSÕES

Esta revisão de escopo permitiu mapear estudos sobre o uso de aplicativos móveis com TNF por pacientes com câncer voltados à QVRS. Esses resultados podem auxiliar na compreensão da relevância dessas tecnologias na autogestão de condições clínicas relacionadas ao tratamento oncológico, com foco na promoção da QVRS nos âmbitos científico e profissional.

Essa temática tem despertado crescente interesse entre a população e os profissionais de saúde. No entanto, ainda existem poucos estudos que avaliam a eficácia das TNF mediadas por aplicativos, o que torna essencial o avanço e o amadurecimento de pesquisas nessa área.

Os achados revelaram que as TNF oferecidas por aplicativos de *smartphones* têm potencial para melhorar a QVRS de pacientes com câncer, além de influenciar variáveis como estresse, ansiedade e depressão. Essa constatação sugere que essas intervenções podem contribuir positivamente como complemento ao tratamento convencional do câncer, promovendo o bem-estar dessa população.

## Data Availability

Os dados de pesquisa só estão disponíveis mediante solicitação.
